# Immediate Catheter Drainage Versus Delayed Drainage in the Management of Infected Necrotizing Pancreatitis

**DOI:** 10.7759/cureus.26485

**Published:** 2022-07-01

**Authors:** Wahidullah Dost, Farzad Qasemi, Wahida Ali, Tahmina Aini, Mohammad Qaher Rasully, Jamaluddin Niazi, Rana Sarhadi jamal, Maseha Sayer, Laila Tul Qadar, Sultan Masoud Shah Afzali

**Affiliations:** 1 General Surgery, Liaquatian Academic & Research Society, Hyderabad, PAK; 2 Cardiovascular Surgery, Punjab Institute of Cardiology, Lahore, PAK; 3 Internal Medicine, Dow University of Health Sciences, Karachi, PAK

**Keywords:** necrotizing pancreatitis, immediate drainage, delayed, drainage, acute pancreatitis

## Abstract

Background: Immediate or delayed catheter drainage of infected pancreatic necrosis remains a subject of debate. The present study aimed to evaluate the optimum timing for drainage in patients with infected necrotizing pancreatitis.

Methods: A prospective, observational study was undertaken at the Department of Surgery, Liaquat University of Medical & Health Sciences (LUMHS), between 1st March 2018 and 6th July 2020. All patients 18 years or older presenting with acute pancreatitis (AP) in whom necrotizing pancreatitis was confirmed or suspected were enrolled in the study. The exclusion criteria included prior intervention for necrotizing pancreatitis. Those who were diagnosed with infected necrotizing pancreatitis were labeled as Group A and Group B. Group A patients underwent immediate catheter drainage (within 24 h of admission) while Group B patients underwent delayed drainage (after 24 h). Clinical outcome variables including complication rate, mortality, length of hospital, and intensive care unit (ICU) stay were collected in a predefined pro forma.

Results: One hundred and thirty patients were enrolled in the study. There were 65 patients in each group. The present study revealed no significant differences in patient outcomes in the immediate drainage group vs. the postponed drainage group. Overall, the mortality rate was 15.38% in Group A while the mortality rate was a little lower in Group B, i.e. 10.77% (p=0.44). The acute onset multiple organ failure was lower in Group A as compared to Group B, however, the difference was statistically insignificant (p=0.08). The rate of wound infection rate was 10.77% and 15.38% in Group A and Group B, respectively (p=0.61).

Conclusion: In the present study, we failed to find any significant difference between the immediate and postponed drainage group in terms of patient outcome. As per current findings, the timing of drainage did not impact the prognosis of patients with necrotizing pancreatitis.

## Introduction

Acute pancreatitis (AP) is a common gastrointestinal presentation to the emergency department worldwide, with an overall mortality rate of 0.5% [1]. Although mild pancreatitis is usually self-limiting, severe pancreatitis can lead to problems like parenchymal/peripancreatic fluid collections and necrosis. Single or multiple organ failure that lasts for more than 48 h and has more than 25% mortality rate falls under the category of severe AP [2-3]. Necrosis of more than 30% of the pancreas confirms that acute necrotizing pancreatitis is present. About 5%-10% of pancreatic cases are having acute necrotizing pancreatitis [2]. The death rate of sterile necrosis is 5%-10% which rises up to 20%-30% once the necrosis becomes infectious [4-5]. Hence, early detection and implementation of appropriate therapy are required.

Pathogenesis involves the unregulated activation of trypsin within the pancreatic acinar cells. Furthermore, a combination of enzymes results in injury to the pancreatic parenchyma which subsequently triggers an inflammatory cascade leading to additional cytokine production, including interleukin IL-1, IL-6 and IL-8, along with tumor necrosis factor α [6]. The endpoint outcome of the deleterious cascade is a systemic inflammatory response syndrome (SIRS). SIRS is defined as loss of vascular tone, decreased systemic vascular resistance, and elevated capillary permeability which results in hypotension. It can further exacerbate and develop respiratory distress syndrome and multiorgan dysfunction syndrome. 

Necrotizing pancreatitis can have a variety of outcomes, including remaining solid or liquefying, remaining sterile, or becoming infected gradually [7]. The initial days following the onset of AP are the most critical, as roughly 15%-25% of individuals are at risk of developing a severe condition [7-8]. Necrosis usually occurs in two stages. A systemic inflammatory response predominates over the first two weeks, which is frequently linked with multiple organ failure, especially during the first 72 h, imparting 50% mortality. The systemic inflammation generally reverts in the second, late phase, which begins 14 days after the onset of symptoms. Around 30% of individuals with necrotizing pancreatitis get affected by infected necrosis [8].

For infected necrotizing pancreatitis, the current suggested treatment is a non-invasive step-up technique that starts with catheter drainage [9]. According to international norms, catheter drainage and antibiotic therapy should be delayed till the peripancreatic necrosis and infected pancreatic have been encased, which takes around four weeks [9]. One advantage of delaying drainage is that antibiotic treatment may eliminate the need for invasive surgery. However, the timings of drainage are, however, still up for debate. Urgent catheter drainage is advised by 45% of professional pancreatologist in a worldwide study following the development of infected pancreatic and peripancreatic necrosis [10-14]. The need for walled-off necrosis in order to perform safe catheter drainage may not be required in this modern age and era, where endoscopic transluminal and noninvasive percutaneous procedures are in use frequently. However, there is no consensus about the most adequate time to intervene in patients with infected necrotizing pancreatitis, and the impact of an immediate or delayed intervention on the overall prognosis is not yet known. Therefore, this study was undertaken to assess the optimum timings for intervention in cases of necrotizing pancreatitis.

## Materials and methods

A prospective, observational study was conducted at the Department of Surgery, Liaquat University of Medical & Health Sciences (LUMHS), between 1st March 2018 and 6th July 2020. After procuring the approval from the ethical committee of LUMHS (LUMHS/REC/155), data acquisition was initiated. A non-probability convenience sampling technique was used for the enrolment of participants. 

The sample size was determined using select statistics online software using a confidence level of 95% and a power of 80%. Reference proportions for the rate of perforation in patients who underwent intervention in less than four weeks and those who underwent drainage ≥ four weeks are 0% and 6%, respectively [14]. A sample size of 123 was determined. 

All patients aged above 18 years who presented with AP in whom necrotizing pancreatitis was confirmed or suspected were enrolled in the study. Patients who have had a previous intervention for necrotizing pancreatitis were excluded. Individuals with infected necrotizing pancreatitis were categorized into two groups, i.e. Group A included patients who underwent catheter drainage immediately (within 24 h of presentation) while Group B included patients who underwent postponed drainage (after 24 h of presentation) [13]. Patients were requested to give informed verbal and written consent after the procedure was narrated to the patients and their attendants. 

If the patient presented in the first two weeks after the diagnosis of AP, the subsequent diagnosis of infected necrosis was made if a positive culture was reported or gas configurations were observed on CT imaging. However, if the patient presented after two weeks of the diagnosis of AP, the diagnosis of infected necrotizing pancreatitis was established by assessing the clinical signs along with the hematological signs, including organ failure, fever, increased levels of C-reactive protein, and leukocyte counts for three consecutive days [11].

All patients who were included in Group A were administered antibiotics and underwent catheter drainage within 24 h after diagnosis (immediate drainage). All patients who were included in Group B underwent delayed catheter drainage and were prescribed antibiotics until walled-off necrosis was noted or when necrotic collections were encapsulated. Image-guided percutaneous catheter drainage was performed after taking the consent of the patients and narrating the risks of the procedure in detail. A needle or catheter was guided through the skin into the necrotized tissue to drain the fluid. 

Demographic and clinical outcome variables including complication rate, mortality, length of hospital, and intensive care unit (ICU) stay were documented. Preoperative and postoperative findings were observed in a predefined proforma. Patients were requested to maintain follow-up visits for up to six months. Socio-demographic variables including age, gender, socioeconomic status, and comorbidities were documented. Moreover, the total length of hospital and ICU stay, mortality rate, severe complications, and cause of AP were reported. 

All data were collected on a structured hard copy questionnaire which was then transferred to the statistical package for social sciences (SPSS version 26, IBM Corp., Armonk, NY). All nominal or ordinal variables were presented as proportions and frequencies while the continuous parameters were presented as mean and standard deviation. Differences between patient outcomes were analyzed using Chi-square test. A p-value of below 0.05 was set as the cut off for statistical significance. 

## Results

Overall, 130 patients (65 in each cohort) were enrolled. Table 1 shows the demographic and clinical characteristics of study participants. The majority of the participants were male with a mean age of 56.8 ± 16.1 years in the immediate drainage group and 54.4 ± 15.5 years in the postponed drainage group. In more than one-half of the population, the cause of pancreatitis was gallstones while in at least 15% of the population, alcohol abuse was the cause of AP. Some 16 (24.6%) patients in Group A and 18 (27.7%) in Group B had diabetes mellitus type II (p = 0.689). 

**Table 1:** Demographic and clinical characteristics of the study population. ICU, intensive care unit

Parameter	Immediate drainage (Group A) (N=65)	Postponed drainage (Group B) (N=65)
Age in years	56.8 ± 16.1	54.4 ± 15.5
Male gender (%)	39 (60.00%)	42 (64.62%)
Cause of pancreatitis (%)		
Gallstones	42 (64.62%)	39 (60.00%)
Alcohol abuse	10 (15.38%)	10 (15.38%)
Other	13 (20%)	16 (24.6%)
Disease severity (%)		
Admitted to ICU	16 (24.62%)	16 (24.62%)
Systemic inflammatory response syndrome	55 (84.62%)	52 (80.00%)
Organ failure	16 (24.62%)	10 (15.38%)
Multiple organ failure	10 (15.38%)	7 (10.77%)

Patient outcomes are shown in Table 2. The study revealed no significant differences in patient outcomes in Group A versus Group B. Overall, the mortality rate was 15.38% in Group A while 10.77% in Group B (p=0.435). The acute onset multiple organ failure was lower in Group A as compared to Group B, however, the difference was statistically insignificant (p=0.08). The rate of wound infection rate was 10.77% in Group A and 15.38% in Group B, respectively (p=0.609). The duration of hospital stay, as well as ICU stay, were significantly lower in patients who underwent immediate drainage with a p-value of 0.0054 and 0.0497, respectively. 

**Table 2:** Comparison of the patients' outcomes between Group A and Group B. ICU, intensive care unit

Patient outcomes	Immediate drainage (Group A) (N=65)	Postponed drainage (Group B) (N=65)	p-value
Mortality (six months postoperative)	10 (15.38%)	7 (10.77%)	0.435
Acute onset organ failure	16 (24.62%)	13 (20.00%)	0.399
Pulmonary dysfunction	7 (10.77%)	10 (15.38%)	0.435
Cardiovascular dysfunction	13 (20.00%)	13 (20.00%)	1
Kidney dysfunction	3 (4.62%)	7 (10.77%)	0.187
New-onset multiple organ failure	4 (4.62%)	10 (15.38%)	0.08
Hemorrhage	10 (15.38%)	13 (20.00%)	0.491
Perforation	7 (10.77%)	7 (10.77%)	1
Pancreaticocutaneous fistula	7 (10.77%)	7 (10.77%)	1
Wound infection	7 (10.77%)	10 (15.38%)	0.609
Length of hospital stay (days)	12.9 ± 7.5	16.7 ± 7.8	0.0054
Length of ICU stay (days)	7.3 ± 2.2	8.1 ± 2.4	0.0497

## Discussion

A severe consequence of AP is infected necrotizing pancreatitis with mortality rates of up to 40% [11-12]. Catheter drainage has been suggested in the latest clinical practice guidelines as the most effective mode of treatment, even when the disease is still in its initial phase [13].

Castillo et al. report that a decrease in mortality rate, complications, and follow-up surgical procedures is associated with closed packaging and drainage of pancreatic necrosis. Even though it is preferable to wait until the necrosis is completely defined before intervening, delaying further than the fourth week provides no further benefit [12].

In line with the current study, a multicenter study conducted by Boxhoorn et al. failed to report any statistically relevant difference in complication rates in immediate catheter drainage as compared to the delayed drainage technique in patients with diagnosed infected necrotizing pancreatitis. However, mortality was a little greater in patients who underwent immediate drainage versus those who underwent delayed drainage (13% vs. 10%) with a relative risk of 1.25 [13]. Moreover, the study also concluded that the mean number of interventions was lower in the postponed intervention group as compared to the immediate drainage group (2.6 vs. 4.4) [13]. 

In contrast to the current findings, some evidence suggests that postponing drainage yields better patient outcomes [14-15]. A retrospective study analyzed the outcomes of infected necrotizing pancreatitis by comparing two groups. One group had 76 patients and they were treated with the endoscopic intervention of necrosis within < four weeks after the onset of pancreatitis, and the second group of 117 patients received the endoscopic intervention of necrosis after four weeks of the onset of AP. Patients of the first group stayed in hospitals for a longer duration than patients treated for more than a month. Patients placed in the immediate intervention group (treated for less than a month), reported a higher incidence of death. (13% vs. 4%, p=0.02) [14]. Similar findings were noted in one more retrospective study. The study compared 38 patients undergoing endoscopic transluminal drainage. Patients treated for less than a month reported having longer durations of stay in hospitals than patients who were given the treatment for more than a month (median, 26 days vs. 6 days; p<0.01). No statistically significant difference was noted in the occurrence of death [15].

Nevertheless, the above-mentioned studies were both limited by the fact that the patients were retrospectively assessed which hinders any legit interference from the findings. In a more comprehensive study, a completely different stance has been offered [16]. Necrosectomy, done within the first two to three days after the commencement of AP is reported to be associated with an increased morbidity and mortality rate than necrosectomy done after the delay of at least 12 days [16].

In another study by Chantarojanasiri et al., it was found that the rate of complications was 25 % in the early drainage group while only 13% in the postponed drainage groups. Two patients died (one in the immediate intervention group while the other in the postponed drainage group) due to multiorgan failure. The fact that the collection was encapsulated and adhered to the gastrointestinal tract leads to the inference that drainage performed within a month after the development of AP is more viable [17]. Just this year, a systematic review has been published evaluating the clinical outcome of pancreatic necrosis in immediate vs. delayed intervention groups [18]. Several hospital-based studies including a total of 742 patients with infected pancreatic necrosis requiring intervention were studied. Of the 742 patients, 321 underwent immediate intervention and 421 underwent delayed intervention. It was observed that the immediate drainage did not raise the in-hospital mortality (p=0.06), however, patients in the immediate group did have a significantly prolonged hospital stay than the delayed intervention [18], which is contradictory to our study (Table 2). 

Infected necrotizing pancreatitis can be a devastating, life-threatening disease and prompt diagnosis and planning of intervention strategy is extremely important. In the present study, we faced certain limitations. For instance, one limitation was the small and undiversified sample size. The findings of the current study cannot be generalized to a larger population. Further research and exploration of the subject is indeed warranted. 

## Conclusions

In the present study, we could not find any statistically relevant differences in the patient outcomes between immediate and postponed drainage groups. Therefore, the timing of drainage does not impact the patient outcomes in necrotizing pancreatitis. We recommend that longitudinal studies should be conducted in multiple settings to deduce a more accurate conclusion regarding the optimum time for intervention in patients who present with necrotizing pancreatitis.
